# Overexpression of *CD73* is associated with recurrence and poor prognosis of gingivobuccal oral cancer as revealed by transcriptome and deep immune profiling of paired tumor and margin tissues

**DOI:** 10.1002/cam4.6299

**Published:** 2023-07-01

**Authors:** Ankita Chatterjee, Amrita Chaudhary, Arnab Ghosh, Pattatheyil Arun, Geetashree Mukherjee, Indu Arun, Arindam Maitra, Nidhan Biswas, Partha P. Majumder

**Affiliations:** ^1^ National Institute of Biomedical Genomics Kalyani India; ^2^ John C. Martin Centre for Liver Research and Innovations Kolkata India; ^3^ Tata Medical Center Kolkata India; ^4^ Indian Statistical Institute Kolkata India

**Keywords:** biomarker, CD73, prognosis, recurrence, immune contexture

## Abstract

**Background:**

For various cancers, differences in response to treatment and subsequent survival period have been reported to be associated with variation in immune contextures.

**Aim:**

We sought to identify whether such association exists in respect of gingivobuccal oral cancer.

**Materials and methods:**

We performed deep immune profiling of tumor and margin tissues collected from 46 treatment naïve, Human Papillomavirus (HPV) negative, patients. Each patient was followed for 24 months and prognosis (recurrence/death) noted. Key findings were validated by comparing with TCGA‐HNSC cohort data.

**Results:**

About 28% of patients showed poor post‐treatment prognosis. These patients exhibited a high probability of recurrence even within 1 year and death within 2 years. There was restricted immune cell infiltration in tumor, but not in margin, among these patients. Reduced expression of eight immune‐related genes (IRGs) (*NT5E*, *THRA*, *RBP1*, *TLR4*, *ITGA6*, *BMPR1B*, *ITGAV*, *SSTR1*) in tumor strongly predicted better quality of prognosis, both in our patient cohort and in TCGA‐HNSC cohort. Tumors of patients with better prognosis were associated with (a) lower CD73+ cells with concomitant lower expression level of NT5E/CD73, (b) higher proportions of CD4+ and CD8+ T cells, B cells, NK cells, M1 macrophages, (c) higher %Granzyme+ cells, (d) higher TCR and BCR repertoire diversities. CD73 expression in tumor was associated with low CD8+ and CD4+ T cells, low immune repertoire diversity, and advanced cancer stage.

**Discussion and conclusion:**

High infiltration of anti‐tumor immune cells in both tumors and margins results in good prognosis, while in patients with minimal infiltration in tumors in spite of high infiltration in margins results in poor prognosis. Targeted CD73 immune‐checkpoint inhibition may improve clinical outcome.

## 1 INTRODUCTION

Oral squamous cell carcinoma has a high incidence with concomitant high mortality (https://gco.iarc.fr/today/data/factsheets/cancers/1‐Lip‐oral‐cavity‐fact‐sheet.pdf).^1^, ^2^, ^3^, ^4^ Oral squamous cell carcinoma of the gingivobuccal region (OSCC‐GB) is the most prevalent oral cancer subtype in India; in western countries tongue cancer is the most prevalent.^5^ OSCC‐GB is strongly associated with tobacco chewing. Patients usually present themselves at a late stage (stage IV) with locally advanced disease. Only about 50% of OSCC‐GB patients survive after treatment and about 30% of patients show post‐treatment recurrence.^6^ OSCC patients who were followed‐up for at least 5 years after treatment showed a median survival time of 29 months and 32.7% of the patients showed recurrence between 2 and 96 months.^7^, ^8^

Immune cells in the tumor microenvironment (TME) play a critical role in cancer progression and response to therapy, particularly immunotherapy.^9^ As a tumor grows, circulating immune cells infiltrate into the site of tumor.^10^ The presence of tumor‐infiltrating lymphocytes (TILs) and active immune responses are of prognostic value in oral cancer.^11^ Immune cells in the TME comprise both anti‐tumor immune cells, such as cytotoxic T‐cells, CD4+ T‐ cells, NK cells, and immunosuppressive immune cells, such as regulatory T‐cells, tumor‐associated macrophages (TAMs), myeloid‐derived suppressor cells (MDSCs).^10^ Complex and dynamic interactions of various immune cell types in the TME determine their functional states and regulate the anti‐tumor immunity in cancer, which ultimately creates a tumor‐promoting or tumor‐rejecting environment.^12^, ^13^ Histological assessment of infiltration of immune cells into the sites of solid tumors has revealed three scenarios^10^, ^14^: (a) abundant infiltration into the tumor and surrounding regions, (b) very low infiltration both in the tumor and the surrounding regions, and (c) arrest of infiltration, that is, immune cells reach the site of tumor but do not infiltrate and are stuck in the surrounding region. These distinct immune contextures are observed across cancer types, and patients with these distinct contextures exhibit differences in response to treatment and survival.^14^

Boxberg et al.^15^ have identified immunological parameters that enable prognostication in OSCC, predominantly for tongue cancer, but not for gingivobuccal cancer. However, they did not investigate the immune cell composition of the histologically negative margin (~5 mm from the core of tumor and devoid of tumor cells; henceforth referred to as the “negative/clear margin”^16^; NCI's Definition of Margin, https://www.cancer.gov/publications/dictionaries/cancer‐terms/def/margin). For many cancers, features of the negative margin, in addition to those of the tumor, enhance prognostication.^17^ In TME, the margin refers to the edge or boundary between the tumor and the surrounding normal tissue. The tumor margin is the interface between the tumor and the surrounding normal tissue, and is therefore a critical site for interactions between tumor cells and the immune system.^18^, ^19^ The status of the margin after surgery can be used to predict the risk of tumor recurrence. A clear margin, free of cancer cells, suggests a lower risk of recurrence, while a positive/invasive margin, with the presence of cancer cells, indicates a higher risk of recurrence. The presence of immune cells in the clear margins of tumors can be a good prognostic factor, as it indicates that the immune system has recognized the tumor as foreign and is mounting an attack against it.^19^ Studies have shown that the type and density of immune cells at the margins of tumors can have a significant impact on patient outcomes. For example, high levels of TILs at the margins of colorectal tumors have been associated with improved survival and lower rates of recurrence. Similarly, high levels of CD8+ T cells at the margins of breast tumors have been associated with improved survival.^20^ Overall, the presence of immune cells in the clear margins of tumors is an indication of good prognosis.

The standard mode of treatment of OSCC‐GB is surgery ± chemo−/radio‐therapy. Immunotherapy in oral cancer is now being considered and restrictively administered.^21^ Since only patients with some specific immune contextures respond to immunotherapy,^22^ there is a growing emphasis to identify biomarkers that are predictive of the success or failure of immunotherapy.^23^ For OSCC‐GB, the nature and extent of variability of immune contextures across patients are largely unknown. The objectives of our study, undertaken in a cohort of OSCC‐GB patients recruited at surgery, were to (a) unravel the heterogeneity of immune contextures, and (b) to identify from the immune contextures signatures of good and poor prognosis (assessed by the propensity of recurrence and length of survival after surgical treatment).

## 2 METHODS

### 2.1 Patient characteristics and sampling

This study was approved by the institutional ethics committees of the National Institute of Biomedical Genomics (NIBMG), and the Tata Medical Center (TMC), Kolkata, India. Forty‐six OSCC‐GB patients who had undergone surgery without receiving any therapy prior to surgery at the TMC, Kolkata, India, between November 2017 and October 2019, and provided written informed consent, were included in the study. OSCC‐GB patients below 18 years of age and those who received treatment prior to surgery were excluded. For Human Papillomavirus (HPV) testing, p16 staining was performed in tissue sections using primary (E6H4) mouse monoclonal antibody (RTU, Roche) on the Ventana platform.^20^ A positive test was based on 8th edition of UICC TNM classification and only HPV‐negative patients were selected. Patients were followed up every 2 months for the initial 2 years (i.e., until November 2021) via telephone calls for assessment of general health condition, symptoms, and recurrence. If the assessment indicated any health problem, the patient was advised to contact TMC without delay. For each patient, follow‐up data were collected for at least 2 years with a median follow‐up time of 24.6 months. Overall survival (OS) time of a patient was calculated from the date of recruitment to the date of death or the date of the last follow‐up. Clinical and pathological details of each patient were collected by the hospital. Demographic details and history of tobacco intake, including the form of intake (smoking/chewing), were also collected.^24^ From each patient, fresh tissue samples were collected at surgery from the (a) tumor center (with at least 50% tumor tissue), (b) negative margin (~5 mm from the tumor edge) and (c) adjacent normal mucosa (at least 2 cm away from the tumor margin). Tissues were histologically examined by two pathologists independently and only tissues of patients with concordant reports were included. Grading and staging of the tumor samples were performed according to the WHO classification of tumors and UICC TNM classification at the time of diagnosis.^25^ The presence of tumor cells in the margins was determined pathologically; negative margins were identified as margin tissues without any detectable tumor cells. All patients received standard‐of‐care treatment according to the National Comprehensive Cancer Network (NCCN) guidelines.^24^ Post‐surgery, each patient received similar treatment that comprised combined radiotherapy and chemotherapy; patients differing in post‐surgery treatment strategy were excluded from the study. The surgically resected margins of patients (available for 43 of the 46 patients) assessed were “negative”^26^ (NCI's Definition of Margin, https://www.cancer.gov/publications/dictionaries/cancer‐terms/def/margin). Information on recurrence was collected for the follow‐up period of 24 months.

### 2.2 RNA sequencing and profiling of immune contextures

Collected tumor, margin, and normal tissues (~25 mg) were stored in RNAlater overnight at 4°C and then shifted to −80°C for long term storage. Each tumor tissue, with at least 50% of tumor purity, was collected from near the center of the tumor. Bulk RNA sequencing was done with triad tissue samples from each patient (tumor, negative margin, and adjacent normal), with a target of 100 million reads per sample (Supplementary Methods 1). RNA‐sequence data were processed, after removal of adapter sequences and low‐quality reads, in accord with the TCGA mRNA Analysis pipeline (https://docs.gdc.cancer.gov/Data/Bioinformatics_Pipelines/Expression_mRNA_Pipeline/). Mapping of sequencing reads to the human genome reference sequence hg19 (NCBI 37) was done. Gene counts were determined using the HTSeq tool.^27^ Normalization of reads and test of equality of the levels of expression of genes between groups, after adjusting for differences in depth, were done using the DESeq2 Bioconductor package within the R statistical programming environment.^28^

Deconvolution of the expression matrix to estimate the proportions of 22 immune cell types in the samples, after gene length normalization, was done using CIBERSORT^29^ (https://ciberfort.stanford.edu/), that uses gene expression “signature matrix” of 547 genes to assess presence of immune cells from bulk tissue specimens (Supplementary Methods 2). Immune activity in the tumor was assessed by curating a list of 2039 IRGs from five different sources (CRIatlas, MSigDB C7 signature [www.gsea‐msigdb.org/gsea/msigdb/], InnateDB,^30^ Immport [www.immport.org], and literature survey^22^, ^31^).

### 2.3 Immunohistochemistry

From each formalin fixed paraffin embedded tumor tissue block, ~3 μm‐thick sections were prepared and single‐marker immunohistochemistry (IHC) was performed in a Bond Max Automated Immunohistochemistry Vision Bio‐system (Leica Microsystems GmbH) using standard protocols.^20^ Briefly, the sequential steps in IHC were: antigen retrieval, addition of primary antibody, application of a secondary antibody that binds the primary antibody, and addition of a detection reagent to localize the primary antibody. The immune markers used were CD20, CD3, CD4, CD8, CD45RA, CD45RO, Granzyme B, and CD73. The detailed process was mentioned in Supplementary Methods.

### 2.4 Estimation of immune repertoire diversity from bulk RNA‐seq data

Reconstruction and estimation of immune repertoires from bulk RNA‐seq data (both B‐cell repertoire [BCR] and T‐cell repertoire [TCR]) were done using TRUST4 algorithm.^32^ The algorithm extracts reads mapped to TCR and BCR gene segments (using the human reference genome sequence hg19), assembles and annotates reads mapping to consensus contigs deposited in the international ImmunoGeneTics (IMGT) database to identify V, J, and C genes (www.imgt.org).^33^

### 2.5 Comparison with the HNSC cohort of TCGA

We used the transcriptome data of the HNSC cohort in the TCGA database for validation of the prognostic gene signature. GEPIA 2021 (http://gepia2021.cancer‐pku.cn/)^34^ was used to study the association of the expressions of immune‐related genes with disease prognosis in the TCGA‐HNSC cohort (*n* = 460). Protein expression using IHC in the HNSC samples was obtained using Human Protein Atlas (https://www.proteinatlas.org/humanproteome/pathology/head+and+neck+cancer). Correlation between the prognostic genes and proportion of immune cells was studied using TIMER2.0 (http://timer.comp‐genomics.org/).^35^

### 2.6 Statistical analysis

Differences in the expression of genes between groups were identified using the DEseq2 R Bioconductor package.^28^ To identify clusters of patients with similar patterns of gene expression, we used Ward's method of hierarchical clustering on the matrix of Euclidean distances, calculated based on normalized gene expression, between pairs of patients. Principal components analysis (PCA) was performed to identify whether different tissue types can be differentiated using expression levels of IRGs (Figure S1). Distributions of categorical clinicopathological features were compared between groups by chi‐squared or Wilcoxon test, as appropriate. Stepwise discriminant analysis was performed to identify gene signatures that may be used to classify patients into distinct prognostic subgroups. Overall survival distributions of the patients in identified subgroups were compared using the Kaplan–Meier method and the log‐rank test. ROC curves were constructed to test the sensitivity and specificity of prognosis using the identified signature genes. Cox proportional hazards model was used to evaluate the effect of gene expression on survival and disease‐free survival. Mann–Whitney *U* test was used to compare the fractions of immune cell types or the distributions of expression levels of proteins assessed by IHC between any two subgroups. Multivariate survival analyses, controlling for stage, age, gender were done to identify the association of immune‐related gene expression in tumor with prognosis. The Benjamini–Hochberg (BH) method was used to adjust *p*‐values for multiple testing. All tests performed were two‐sided, and a *p*‐value of less than 0.05 was considered as significant, unless stated otherwise. Data were analyzed using R (V.4.0.2).

## 3 RESULTS

### 3.1 Significantly different proportions of immune cell types were present in the tumor core compared to surrounding margins

**FIGURE 1:**
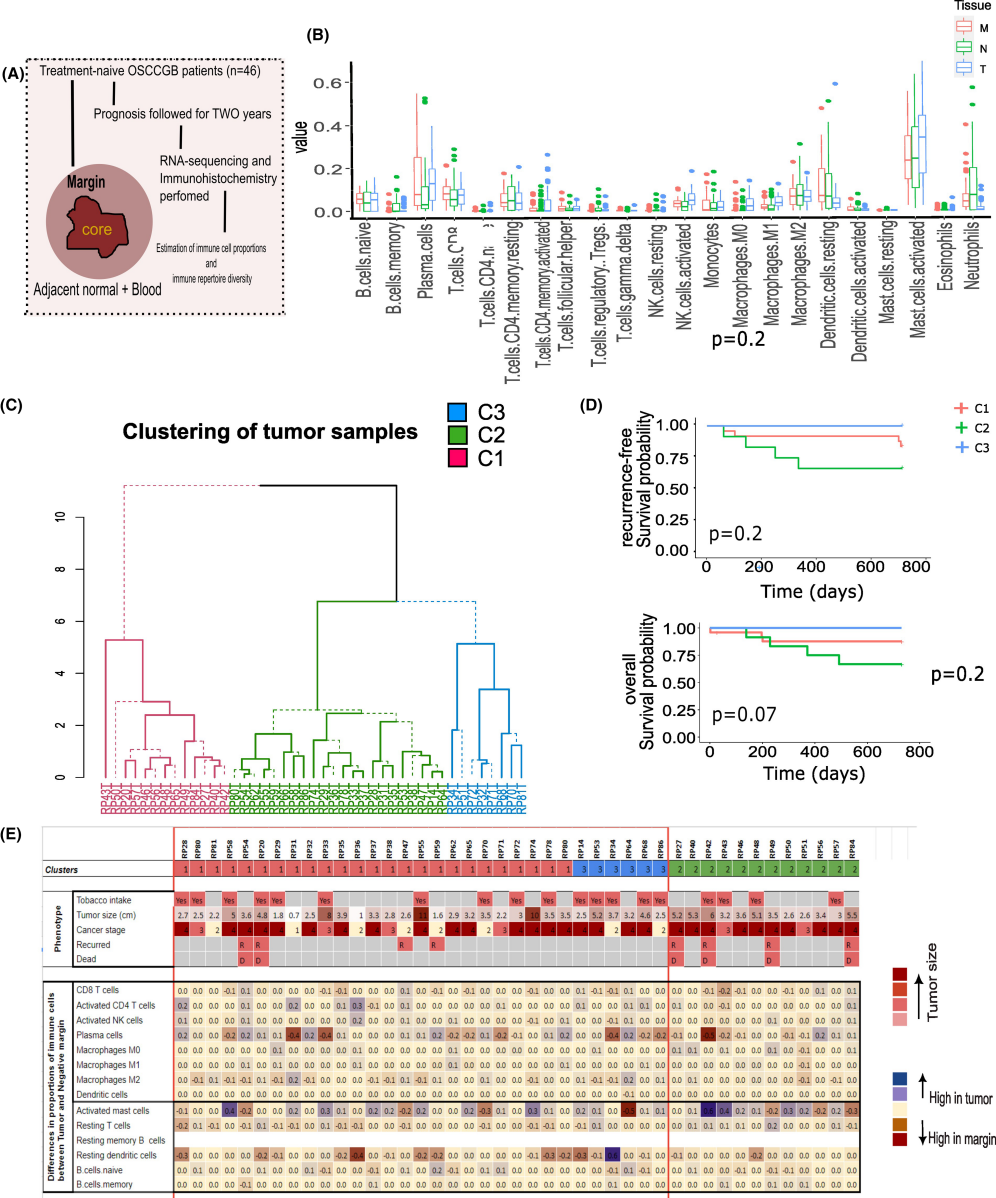
Clustering of oral squamous cell carcinoma of the gingivobuccal (OSCC‐GB) patients, based on the expressions of immune‐related genes. (A) Schematic diagram explaining the study design. Tumor, negative margins, and adjacent normal tissue samples were obtained from 43 OSCC‐GB patients and bulk transcriptomic data was generated. The patients were followed up for 2 years to track disease prognosis. (B) Box plots of data on proportions of 22 immune cell types in tumor, margin, and normal tissue. (C) Hierarchical clustering of patients depending on the expressions of 544 immune‐related genes. Three separate clusters of patients were identified—C1 (red), C2 (green), and C3 (blue). (D) Differential disease prognosis (recurrences and deaths) of the three clusters post‐surgery. C2 patients showed the worst prognosis. (E) Tobacco usage and clinical profiles of the 43 OSCC‐GB patients belonging to three clusters and the distribution of proportions of immune cell types in the tumor centers of each patient. The proportions of immune cell types were estimated using CIBERSORT from the bulk tumor specimen.

Most OSCC‐GB patients were middle‐aged (mean age = 56.7 years), male (83.7%), tobacco users (53.5%; smokers = 13.9% and 46.5% chewed tobacco) and had presented themselves with stage IV disease (60.9%) (Table 1; Figure 1A). Among the 46 patients, 48.8% were T4, 25.5% were T3, 20.9% were T2 and 4.6% were T1. Nodal metastasis was observed among 48.8% of the patients. All tumors were HPV negative. During the follow‐up period of 24 months after surgery, eight (18.6%) patients showed local recurrence, among whom six died within the follow‐up period. Worst pattern of invasion (WPOI)—types 4 (intermediate) and 5 (high) were pooled because of limitation of the total sample size of this study—was observed among 20.9% of the patients. Distant metastasis was observed in only two patients.^36^

**TABLE 1:** Characteristics of recruited patients and of three homogeneous subsets of patients.

	All patients (*n* = 46)	GPC (*n* = 33)	BPC (*n* = 13)	*p*‐value**
% male	83.7	83.8	83.3	0.96
Mean age ± SD (years)	56.6 ± 10.4	58.16 ± 9.16	52.8 ± 10.8	0.4
Stage				
% stage IV	65.1	58.1	83.3	0.2
% stage III	13.9	12.9	16.6	
% stage II	18.6	25.8	0.0	
% stage I	2.3	3.2	0.0	
T‐stage				
% T4	48.8	41.9	66.7	0.37
% T3	25.5	25.8	25.0	
% T2	20.9	25.8	8.3	
% T1	4.6	6.4	0.0	
N‐stage				
%N3	16.2	16.1	16.6	0.78
%N2	16.2	16.1	16.6	
%N1	16.2	12.9	25.0	
%N0	51.1	54.8	41.6	
% recurred during follow‐up period	17.3	12.1	30.7	0.16
% dead during follow‐up period	13.0	9.1	23.1	0.4
% node metastasis observed	48.8	45.2	58.3	0.6
% WPOI observed	20.9	25.8	8.3	0.4
% smokers	13.9	12.9	16.6	0.9
% chewed tobacco	46.5	48.3	41.6	0.95

Abbreviations: BPC, bad prognosis cluster; GPC, good prognosis cluster; WPOI, worst pattern of invasion.

** *p*‐value corresponds to the test of equality of proportions or mean values among the groups (with non‐zero proportions).

Estimates of abundance of 22 different immune cell types in the tumor, negative margin and the normal tissues in the pooled set of samples are presented in Figure 1B. The most abundant cell types in OSCC‐GB tumors were activated mast cells (mean %: 31%), plasma cells (13%), and M2 macrophages (7%). We observed that both the tumors and the margins had higher proportions (most statistically significant) of plasma cells, memory activated CD4+ T cells, follicular helper T cells, M1 macrophages, compared to adjacent normal tissues (Table S2). Tumor centers had significantly higher proportions of memory activated CD4+ T cells, follicular helper T cells, and M0 and M1 macrophages, compared to both the negative margins and the adjacent normal tissues. Proportions of resting dendritic cells were higher in negative margins compared to tumor tissues. This is indicative of infiltration of immune cells in the TME (Figure 1B; Table S1). No significant associations between the proportions of immune cells in tumor and WPOI were observed; although tumors with presence of WPOI had lower proportion of CD8 T cells and M1 macrophages while higher proportions of M2 and activated dendritic cells (Figure S1).

### 3.2 Expressions of immune‐signature genes in tumor stratified good‐prognosis and poor‐prognosis clusters of OSCC‐GB patients

Differential gene expression (DGE) analysis identified 544 of the 2039 IRGs to be significantly differentially expressed (Benjamini–Hochberg corrected *p*‐value <0.05) in the tumor compared to the adjacent normal tissue (Table S1). Among these 544, 193 IRGs (35.4%) were differentially expressed in the tumor compared to negative margins. Of these, the most significant IRGs were *FABP3*, *CASQ1*, *IL17D*, *ADIPOQ*, *EDN3* and the most significantly enriched pathway was “Cytokine‐cytokine receptor interactions” (Figure S2).^37^, ^38^ Only 15 IRGs (2.7%), affecting “growth factor activity” (assessed using DAVID^37^; https://david.ncifcrf.gov/), were differentially expressed (*p* = 6.6 × 10^−3^) in the margins compared to the adjacent normal tissues (Table S1). Immunologically, therefore, margins and normal tissues were similar, but tumors were distinct from the respective negative margins and normal tissues (Figure S2).

To investigate whether the levels of expression in the tumor of the 544 significantly differentially expressed IRGs may help identify subgroups of patients with similar tumor gene‐expression, we carried out a cluster analysis (see Sections 2 and 2.6). We identified three clusters among the 46 patients, each cluster with a similar pattern of gene expression among the patients comprising the cluster (Figure 1C); cluster‐1 (C1; 26 patients), cluster‐2 (C2; 13 patients), and cluster‐3 (C3; 7 patients), but dissimilar with patients belonging to a separate cluster. Patients in C2 were those with bad prognosis (bad prognosis cluster; BPC) since (Table 1; Figure 1D): (a) during the follow‐up period of 24 months, 33.3% of these patients had recurrence compared to 16% and 0% of patients in C1 and C3, respectively, and (b) a significantly (*p* < 0.05) lower (66.7%) proportion of patients survived for at least 2 years after surgery compared to 92% and 100% of patients in C1 and C3, respectively. Thus, patients in C1 and C3 exhibited much better prognosis; we designate the pool of patients in these two clusters to belong to a good prognosis cluster (GPC).

Comparing the distributions of the immune cell‐types of patients with good (GPC) and bad (BPC) prognoses (Figure 1E; Table S1), we found that (1) in both tumor and the negative margin, resting dendritic cells were significantly higher in the GPC than in the BPC, while activated mast cells were significantly higher in the BPC compared to the GPC, (2) naive B cells, plasma cells, follicular helper T cells were significantly higher in the GPC tumors compared to the BPC tumors, (3) among the GPC patients, activated CD4+ T cells, activated NK cells, and activated M1 macrophages were significantly higher in the tumor than the negative margins, while resting CD4+ T cells, resting dendritic cells and neutrophils were significantly lower in the tumor and (4) among the BPC patients, resting dendritic cells were significantly higher and follicular helper T cells, M0 macrophages and neutrophils were significantly lower in the tumor tissues compared to the negative margins. Thus, except for resting dendritic cells and activated mast cells, the immune composition of the negative margins of GPC and BPC patients were similar. In the tumor, however, the profiles of immune cell types were vastly different in patients with bad prognosis than those with good prognosis among whom there was higher infiltration of anti‐tumor immune cells (Figure 1E).

For validation, we performed IHC on tumors of a subgroup of 33 (GPC, *n* = 23; BPC, *n* = 7), of the 43 patients on whom sufficient tissue was available for performing IHC. Consistent with our findings of CIBERSORT analysis, the percentage of CD20+ B cells was found to be significantly (*p* = 0.037) higher in tumors of GPC patients (median = 5.4%), compared to those in BPC (3%) (Figure S3). The proportion of CD45RA+ cells (activated T cells) was significantly (*p* = 0.07) higher among patients of GPC (median = 19.05%) than among BPC (10.25%). The percentages of CD4+ T cells (median = 28.65% in GPC vs. 13.5% in BPC) and CD8+ T cells (median = 17.9% in GPC vs. 8.3% in BPC.) were also higher (over two times; although not statistically significant) in GPC than in BPC (Figure S3). Cytotoxic killing of tumor cells by CTLs and NK cells, restrict tumor growth.^20^, ^39^, ^40^ Using immunohistochemistry, we estimated the median percentages of cells expressing cytotoxic markers, GranzymeB, and Perforin. These were, for GPC and BPC tumors, respectively (a) for Granzyme+ cells: 4.2% and 0.65% (*p*‐value for significance of difference = 0.016); (b) Perforin + cells: 2% and 0.7% (Figure S3).

### 3.3 Expression levels of *CD73* and *ITGA6* were associated with poor prognosis

**FIGURE 2:**
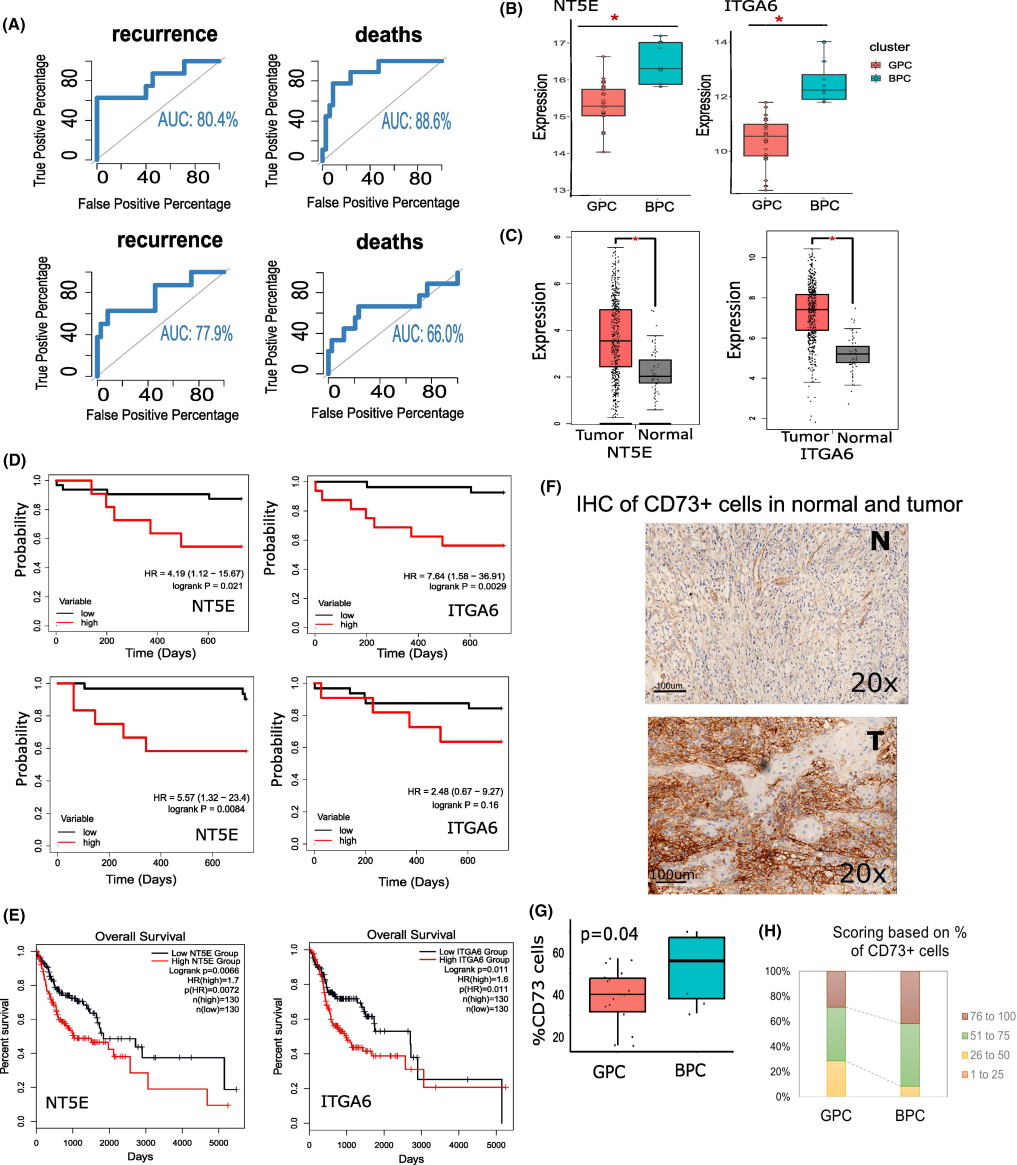
Overexpression of CD73 and ITGA6 and oral squamous cell carcinoma of the gingivobuccal (OSCC‐GB) prognosis. (A) Area under the curves showing the sensitivity and specificity, to predict prognosis (recurrences and deaths) of eight immune related genes (upper panel) and two genes (CD73 and ITGA6) (bottom panel). (B) Significantly higher expression of the two immune‐related genes (IRGs) in BPC tumors, compared to GPC tumors. (C) Over‐expression of the two IRGs in HNSC tumors of the TCGA cohort, compared to the adjacent solid tissue normal samples. (D) Poor prognosis of tumors with higher expressions of the two IRGs in our cohort (top panel: recurrence; bottom panel: deaths), and (E) in the TCGA‐HNSC cohort. (F) Results of immunohistochemistry; % cells positive for CD73 immune. Representative IHC images at 20× magnification are shown—in normal tissue (top panel) and in tumor center (bottom panel). (G and H) Percentages of CD73+ cells with frequencies in various score‐intervals of IHC staining for GPC and the BPC tumors. BPC, bad prognosis cluster; GPC, good prognosis cluster.

**FIGURE 3:**
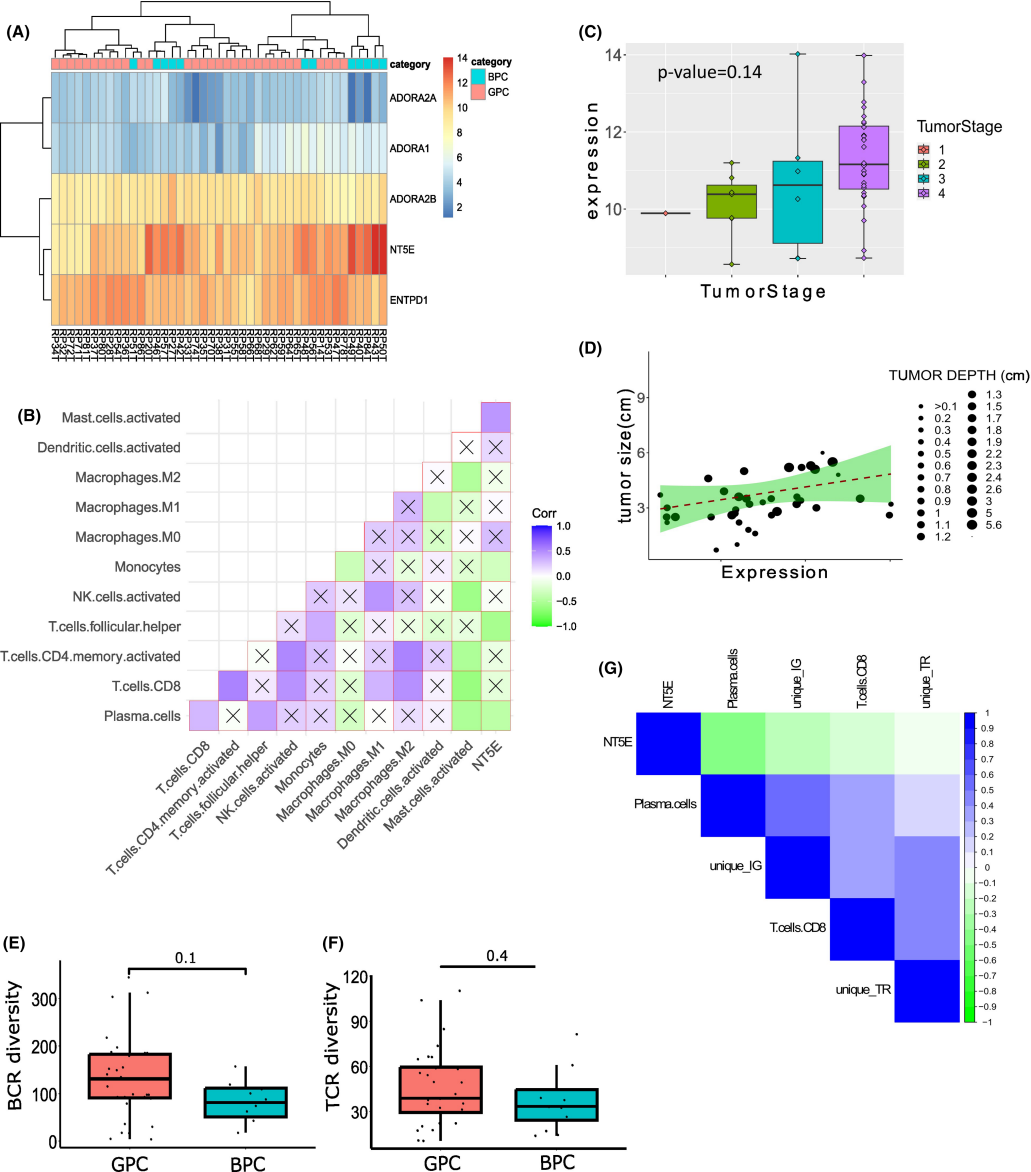
Negative association of CD73 with anti‐tumor immune cell infiltration. (A) Heatmap showing normalized expressions of five genes (rows) in the adenosine metabolism for each tumor sample (columns). (B) Correlation plot among the immune cells and normalized CD73 expression in tumor. “*X*” = the correlation between the parameters were not significant; filled colored boxes signified significant correlation, *p*‐value <0.05. (C) Boxplots showing expression of CD73 across tumor stages. (D) Correlation between CD73 expression and tumor size. Boxplots showing higher BCR (E) and TCR (F) diversity in GPC compared to BPC. (G) Correlations among the immune repertoire and CD73 expression. BCR, B‐cell repertoire; BPC, bad prognosis cluster; GPC, good prognosis cluster.

Stepwise discriminant analysis based on the expression levels of 544 IRGs, identified that expressions of eight genes (*CD73*, *THRA*, *RBP1*, *TLR4*, *ITGA6*, *BMPR1B*, *ITGAV*, *SSTR1*) were significantly lower in patients with good prognosis (GPC) compared to the remaining patients (BPC) (Table S3). We, therefore, investigated whether data on these eight genes could provide a reasonable classification of patients into two groups with good and bad prognosis. We found that the expressions of the eight IRGs provided >80% correct classification of patients to GPC and BPC (Figure 2A). Among the eight IRGs, expression levels of two genes—*ITGA6* and *CD73* were (1) overexpressed in tumors in BPC compared to tumors in GPC (Figure 2B), (2) overexpressed in tumors compared to normal tissues, both in our cohort and TCGA‐HNSC cohort (Table S2; Figure 2C), (3) patients with higher expressions of the two genes showed significantly worse prognosis, both in our cohort (Figure 2D) and in TCGA‐HNSC cohort (Figure 2E) and (4) The expressions of *ITGA6* and *CD73* showed a correct classification of ~70% between the good and poor prognosis (Figure 2A).

Using IHC stained for CD73+ cells (Figure 2F), we found that (1) the mean percentage of CD73+ cells were higher in BPC (mean 42.4%) tumors compared to those in GPC (mean 28%), consistent with our transcriptome data (Figure 2G) and (2) %BPC samples with >75% of CD73+ cells was higher than %GPC tumor samples (Figure 2H).

### 3.4 *CD73* expression is negatively associated with anti‐tumor immune cells and better prognosis

*CD73*, also known as Ecto‐5′nucleotidase, controls extracellular adenosine generation from AMP. Recent studies showed that extracellular adenosine regulates activities of immune cells in the TME.^41^ To understand the role of adenosine metabolism pathway in OSCC‐GB prognosis, differential expressions of the five genes (*CD73*, *ENTPD1/CD39*, *ADORA1*, *ADORA2A*, and *ADORA2B*) were observed across the samples. Both *CD73* and *ADORA2A* genes were over‐expressed in the BPC tumors (Figure 3A).

*CD73* expression in tumor showed a significant negative correlation with CD4+ helper T cells and Plasma cells (Figure 3B). Although, not significant, negative correlations between *CD73* gene expression and CD8+ T cells, CD4+ activated T cells and activated NK cells were observed in the tumor samples. Using TIMER 2.0,^35^ we found significant negative correlations between the expressions of *ITGA6* and *CD73* with CD8+ T cell and plasma cell infiltration in tumor (Figure S4). Expression of *CD73* was observed to be the highest among the stage IV cancer samples (Figure 3C) and was positively correlated with tumor size and tumor depth (Figure 3D), thus confirming its association with cancer severity.

To study the clonal expansion of antigen‐specific T cells and B cells, we reconstructed the immune repertoire from bulk RNA‐seq data.^32^ Clonotypes were constructed from the reads aligned to the IMGT database.^33^, ^42^, ^43^ Of the total number of sequencing reads, ~0.8% mapped to immune repertoire genes (including the V, D, J, and C gene segments of the T cell and B cell receptor gene loci). Of the constructed clonotypes, >90% were BCRs and ~10% comprised TCRs (Figure S5). The total number of unique clonotypes, clonotype “richness,” in tumors ranged from as low as 10 to ~19,000 for IG/BCR and to ~400 for TCR (Figure S5). The diversities of both BCR clonotypes and TCR clonotypes (measured by the Inverse Simpson index^42^) were higher in tumors, but not in the negative margins (Figure S5), of patients belonging to GPC compared to those of BPC (Figure 3E,F), indicating higher T‐cell and B‐cell functionality of GPC tumors than of BPC. Significant negative correlations between *CD73* gene expression and the number unique clonotypes (both BCR and TCR) were observed (Figure 3G). All the evidence pointed to the negative association of *CD73* gene expression with anti‐tumor immune infiltration in OSCC‐GB cancer.

### 3.5 Prognosis is poor with altered ECM‐receptor interaction

*ITGA6* gene is an integrin molecule expressed on epithelial cells, which regulates cell–cell adhesion and maintaining ECM‐receptor interaction. Increased levels of *ITGA6* can promote tumor susceptibility and progression.^44^ In addition, *ITGA6* is directly regulated by hypoxia‐inducible factor (HIF) and high *ITGA6* expression enhances invasion of cancer.^44^ We observed significant over expression of *ITGA6* gene in tumor compared to adjacent normal (Figure 2B) and in BPC tumors compared to GPC tumors (Figure 2C). Additionally, we also noted that overexpression of *ITGA6* gene was associated with poor prognosis (Figure 2D). The expression of *ITGA6* gene was negatively associated with anti‐tumor immune cells, such as plasma cells and CD8+ T cells and most importantly with activated NK cells (Figure S5A). Gene‐set enrichment analysis showed that genes in ECM‐receptor interaction pathway (including *ITGA6*), were overexpressed in BPC tumors compared to GPC tumors (Figure S6B,C). We further investigated the relationship between the expressions of genes in ECM‐receptor interaction pathway with the presence of immune cells in the tumor by calculating a pathway‐specific activation score.^45^ The magnitude of the score increases with simultaneous increase of gene expression and higher positive coefficient, the pathway score is positive ranging from 120 to 200 (Figure S6D). “ECM‐receptor interaction” score was also significantly higher in the BPC tumors and stage IV tumors (Figure S6E). A significant negative correlation with helper T cells and positive correlation with M0 macrophages were observed with pathway‐specific score of “ECM receptor interaction” (Figure S6F).

## 4 DISCUSSION

Intricate crosstalk between malignant cells and infiltrating immune cells and fibroblasts in the TME critically determines the initiation, progression, and therapeutic response in human cancer.^9^, ^12^, ^46^ This study is possibly the first comprehensive investigation on immune profiling of the TME in the most common type of cancer affecting the oral cavity—gingivobuccal oral cancer—in India and southeast Asia. In this study, a triad sampling approach (tumor, negative margin, and normal tissue from the same patient) has been used. The purpose of this study was to discover expression signatures that correlate with the prognosis of the disease.

Transcriptomic studies have shown that the prognosis of cancer differs in patients with variable expression levels of immune‐related genes.^22^ In this study, we identified two clusters of OSCC‐GB patients who showed distinct disease outcomes during a 2‐year follow‐up period after treatment (surgery). These clusters were—Good prognosis (GPC, cluster of patients with almost no recurrence within 1 year of treatment and no death within 2 years) and Bad prognosis cluster of patients (BPC) with high recurrence and low survival. We identified a profile of reduced expression of eight IRGs—*CD73*, *THRA*, *RBP1*, *TLR4*, *ITGA6*, *BMPR1B*, *ITGAV*, *SSTR1*; this signature correctly classified 97% of patients. Among them, *CD73* is a membrane protein involved in nucleotide metabolism and determines lymphocyte differentiation. ATP in the tumors can be degraded by ectonucleotidases, such as *CD73* and *CD39*, to produce free adenosine, that can inhibit immune responses and can promote immune escape of tumor cells.^41^, ^47^, ^48^ *CD73* is known to be associated with immune suppression and poor prognosis in head and neck cancers^47^; *CD73* inhibition may be targeted for immunotherapy.^49^ Integrins are heterodimeric transmembrane cell adhesion receptors which mediate cell‐to‐cell and cell‐to‐extracellular matrix interactions. *ITGA6* is a cell adhesion molecule; upregulation of this gene is associated with cell migration, metastasis, and epithelial‐to‐mesenchymal transition (EMT).^50^

A patient's immune system participates in shaping the TME, either shifting toward a pro‐ or an anti‐tumorigenic milieu. Knowledge of the nature and extent of immune response helps to predict disease‐free survival and the use of adjuvant therapies.^51^ Prognosis of cancer patients is dependent on the density and the diversity of tumor‐infiltrating immune cells in the tumor.^11^ CD4+ helper T cells can facilitate the production of antibodies and phagocytosis by B cells and was shown to be associated with GPC in OSCC.^52^, ^53^ Helper T cells can restrict tumor proliferation and facilitate tumor rejection in oral cancer.^54^ Tumors of patients with GPC had higher presence of CD4+ T cells, CD8+ T cells, B cells, and M1 macrophages compared to those of patients with BPC. Cytotoxic CD8+ T cells have major anti‐tumor activity by destroying tumor cells (Table S3)^20^; these T cells were found to be associated with a GPC in oral cancer.^20^ Our data have also shown negative correlations between the expressions of *CD73* and *ITGA6* genes and the proportions of CD8+ T cells and B cells in the tumor (Table S4). Other immune cells with anti‐tumor effects, including NK cells and M1 macrophages, were also associated with better prognosis of OSCC patients.^55^, ^56^, ^57^, ^58^ The GPC tumors had more activated CD4+ T cells, activated NK cells, M0 and M1 macrophages, compared to negative margins (Table S4). Contrarily, both CD4+ T cells and M0 macrophages were fewer in the BPC tumors compared to the negative margins. Resting CD4+ T cells were more prevalent in the negative margins of GPC patients and were less prevalent in BPC negative margins, compared to the tumors of the corresponding patients. To investigate which of the immune cell type proportions in tumor, margin, and normal tissues significantly predict survival, we carried out logistic regression analyses. However, the results did not reveal any interesting pattern (Table S3) that was statistically significant, possibly because of the limited sample size of this study. Taken together, these features suggest that while the negative margins of patients had similar compositions and levels of infiltration of immune cells irrespective of prognosis, the tumors of patients with GPC contained more antitumor immune cells and tumors of patients with BPC were devoid of anti‐tumor immune cells. We found that tumors of GPC patients were enriched with T cells. It is known that cytolytic T lymphocytes secrete pore‐forming protein perforin and serine protease granzymes, that cause lysis of tumor cells.^59^ Expression of granzyme B was significantly higher in tumors of GPC patients than of BPC patients. It has been previously reported that these cytolytic factors are selectively expressed in oral cancer and predict GPC.^60^ The diversity and richness of immune repertoires including both the BCRs and TCRs were high in the GPC patients that further supports higher immune infiltration and better antigen recognition that can possibly explain better prognosis in these patients.^61^

Using GSEA analysis, we have found that genes involved in “ECM‐receptor interaction” and “focal adhesion,” including *ITGA6*, were upregulated in the tumors of BPC than in GPC. Activation of ECM‐receptor interactions is a known hallmark for EMT.^62^, ^63^ EMT in cancer is associated with expulsion of immune cells thus affecting anti‐tumor immune response in cancer.^62^ EMT was found to be associated with significantly lower infiltration of CD4+ T cells and CD8+ T cells in lung cancer, as we have found in patients with BPC.^64^ In patients with BPC, we also observed upregulated expressions of genes with “growth factor activities.” Previous studies have established that growth factors can induce EMT in cancer.^63^ Thus, EMT seems to play an important role in precipitating or modulating the progression of OSCC‐GB.

In sum, our findings reveal that there is considerable post‐treatment variability in prognosis in gingivobuccal oral cancer. Among patients with GPC, there is high infiltration of anti‐tumor immune cells in tumors and in tumor margins. However, among patients with BPC, while there is high infiltration of immune cells in tumor margins, there is insignificant presence of immune cells in tumors. CD73+ cells are found in significantly larger proportions in tumors with BPC. These cells act as immune checkpoints and targeted inhibition of these cells may improve clinical outcome. We recognize that the number of patients included in our study is somewhat limited; therefore, our results need to be validated in an independent sample.

## AUTHOR CONTRIBUTIONS

**Ankita Chatterjee:** Data curation (lead); formal analysis (lead); investigation (lead); methodology (lead); software (lead); visualization (lead); writing – original draft (lead); writing – review and editing (equal). **Amrita Chaudhary:** Validation (lead). **Arnab Ghosh:** Formal analysis (equal). **Pattatheyil Arun:** Investigation (equal); methodology (equal); project administration (equal); resources (equal); supervision (equal); writing – original draft (equal). **Geetashree Mukherjee:** Data curation (equal); project administration (equal); validation (equal); visualization (equal); writing – original draft (equal). **Indu Arun:** Investigation (equal); project administration (equal); resources (equal). **Arindam Maitra:** Conceptualization (equal); data curation (equal); funding acquisition (equal); investigation (equal); methodology (equal); project administration (equal); resources (equal). **Nidhan Biswas:** Conceptualization (equal); data curation (equal); formal analysis (equal); funding acquisition (equal); investigation (equal); project administration (equal); supervision (equal); writing – review and editing (equal). **Partha P. Majumder:** Conceptualization (lead); formal analysis (equal); funding acquisition (lead); investigation (lead); methodology (equal); project administration (equal); resources (equal); supervision (lead); writing – original draft (lead); writing – review and editing (lead).

## FUNDING INFORMATION

The study was funded by Department of Biotechnology, Government of India as a part of Systems Medicine Cluster project (Grant No. BT/Med‐II/NIBMG/SyMeC/2014/Vol.II).

## CONFLICT OF INTEREST STATEMENT

The authors declare no potential conflict of interest.

## ETHICS APPROVAL AND CONSENT TO PARTICIPATE

The study was approved by the Institutional Review Boards of the National Institute of Biomedical Genomics, Kalyani, India and the Tata Medical Centre, Kolkata, India. All the study participants were recruited in the study with proper informed consent.

## Supporting information


Figure S1:

Figure S2:

Figure S3:

Figure S4:

Figure S5:

Figure S6:

Table S1:

Table S2:

Table S3:

Table S4:

Data S1.

## Data Availability

Data (metadata and raw RNA_seq counts) have been submitted to GEO: GSE213862.
