# Supplementary figures and images for: Overexpression of CD73 is associated with recurrence and poor prognosis of gingivobuccal oral cancer as revealed by transcriptome and deep immune profiling of paired tumor and margin tissues
Source: Cancer Med. 2023 Jul 1;12(16):16774–87. doi: 10.1002/cam4.6299 (PMC10501293; doi:10.1002/cam4.6299)

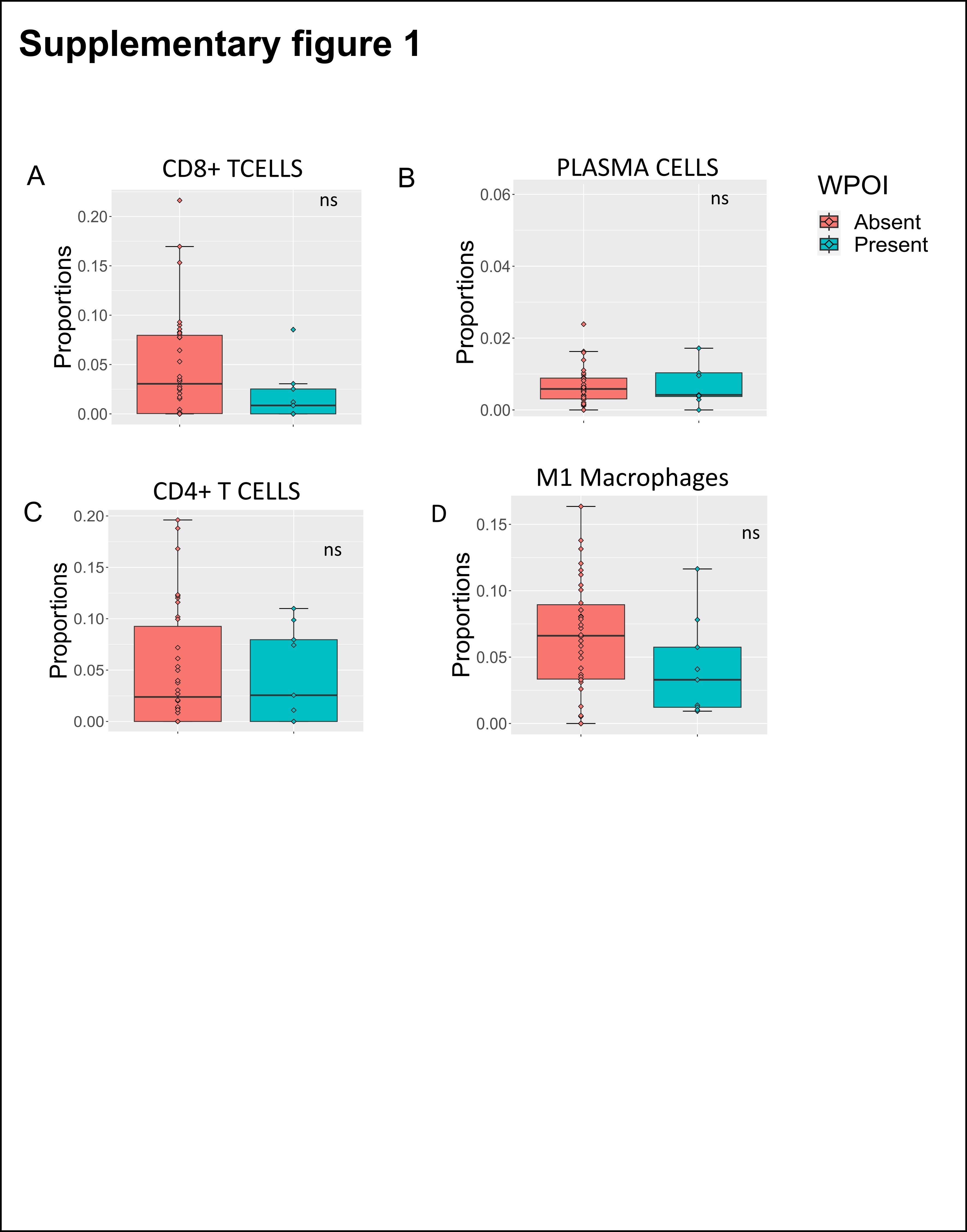

Supplement: Supplementary file 1 — Figure S1: [file CAM4-12-16774-s001.jpg]

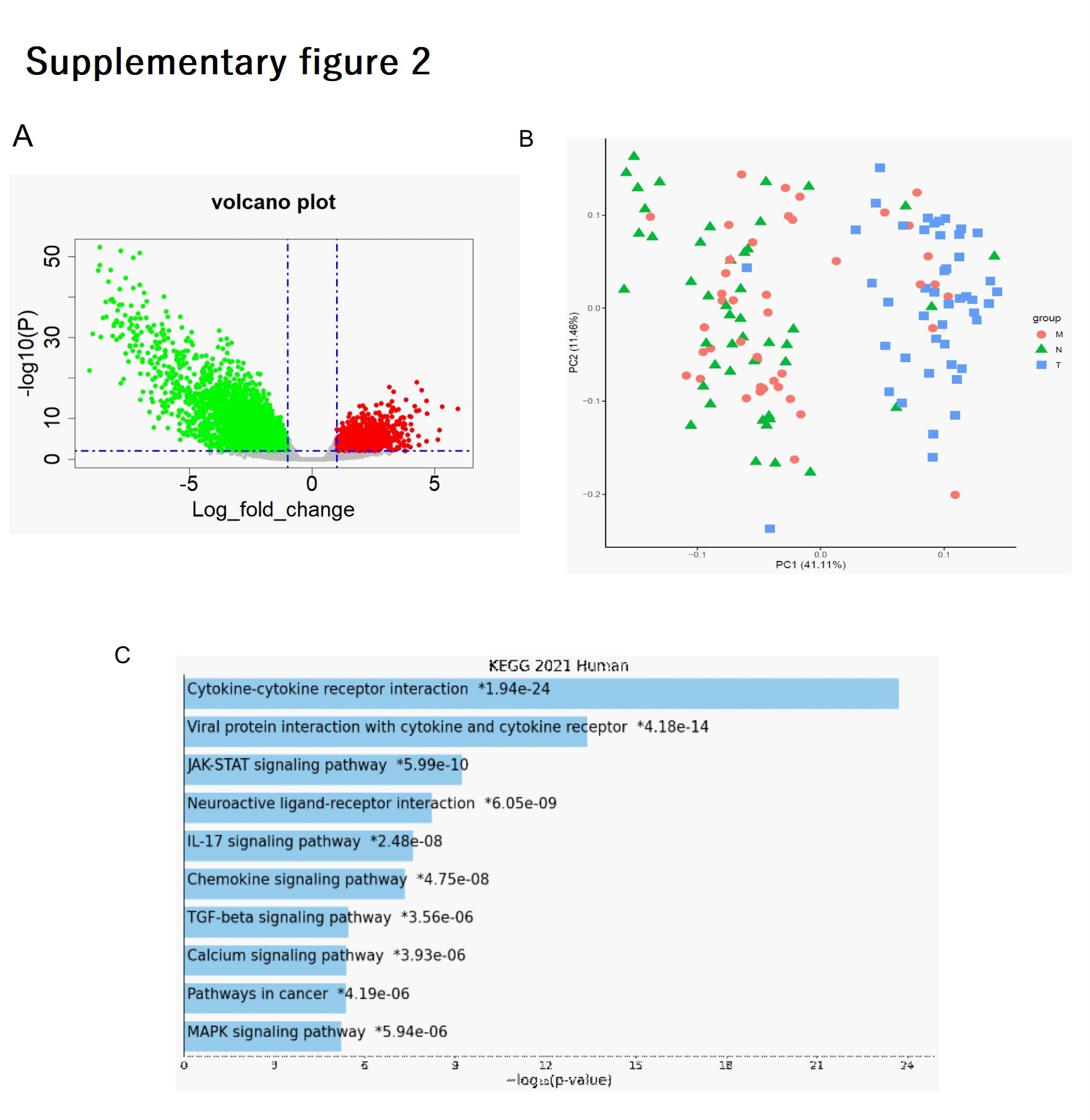

Supplement: Supplementary file 2 — Figure S2: [file CAM4-12-16774-s003.jpeg]

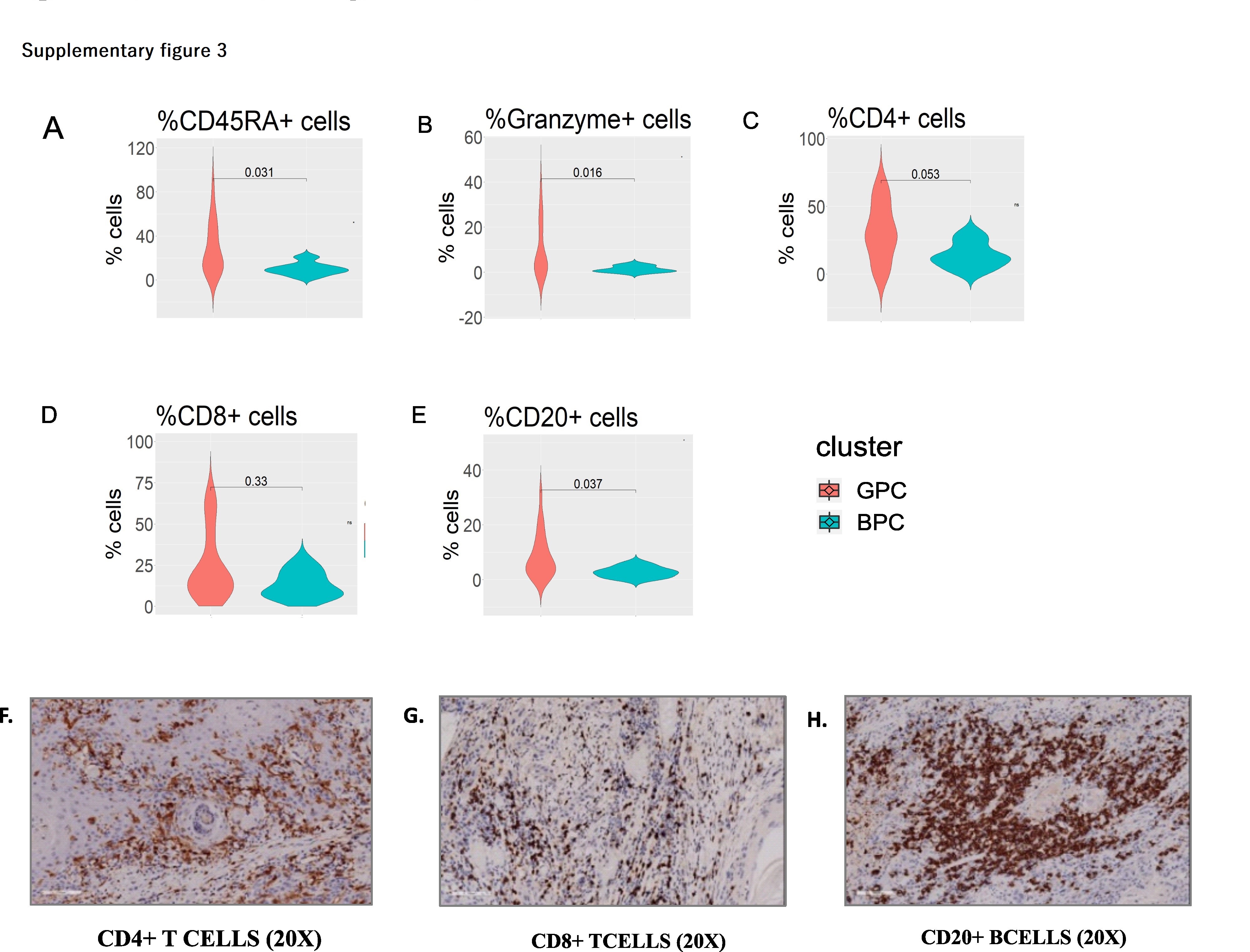

Supplement: Supplementary file 3 — Figure S3: [file CAM4-12-16774-s007.jpg]

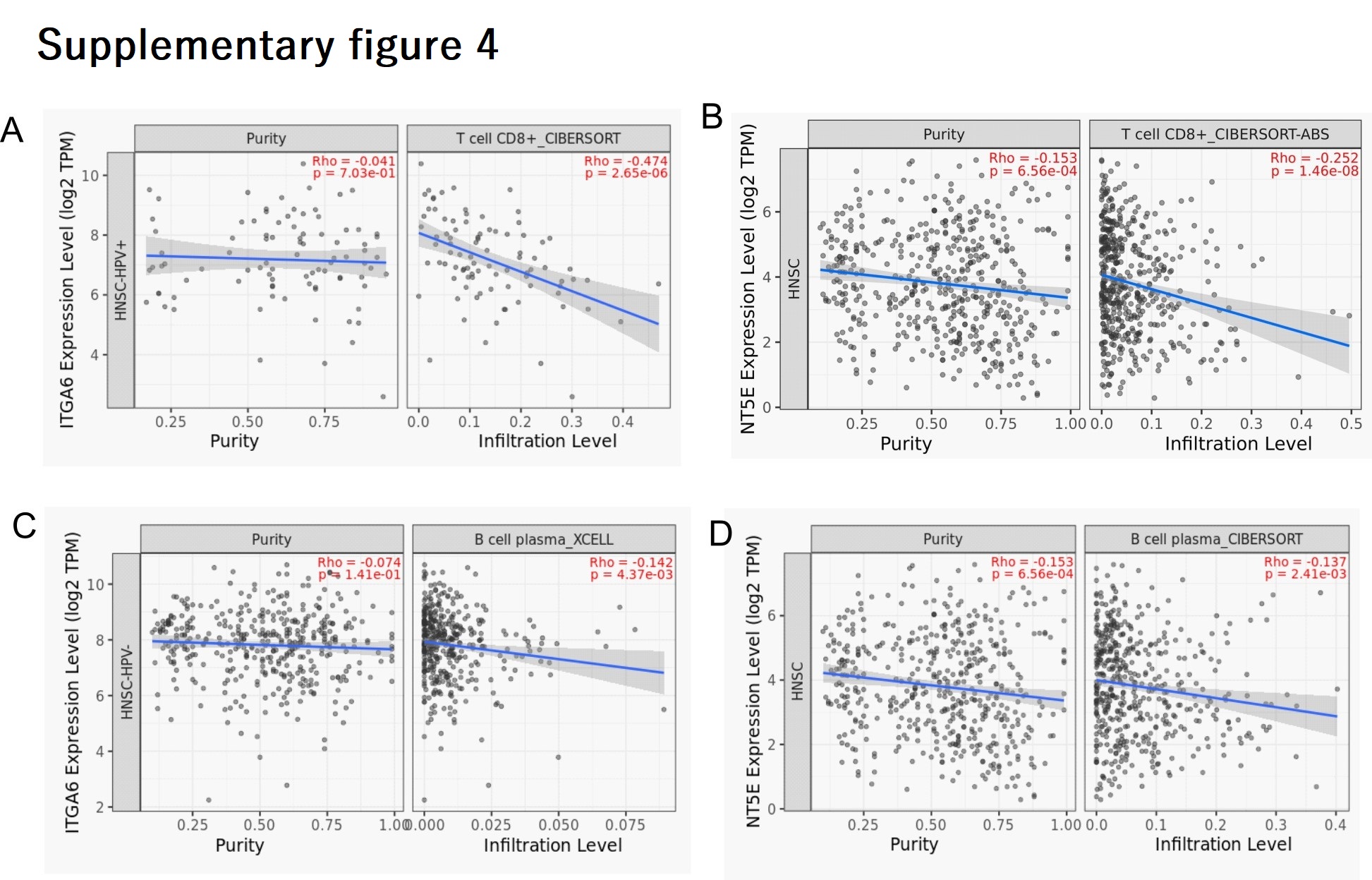

Supplement: Supplementary file 4 — Figure S4: [file CAM4-12-16774-s004.jpeg]

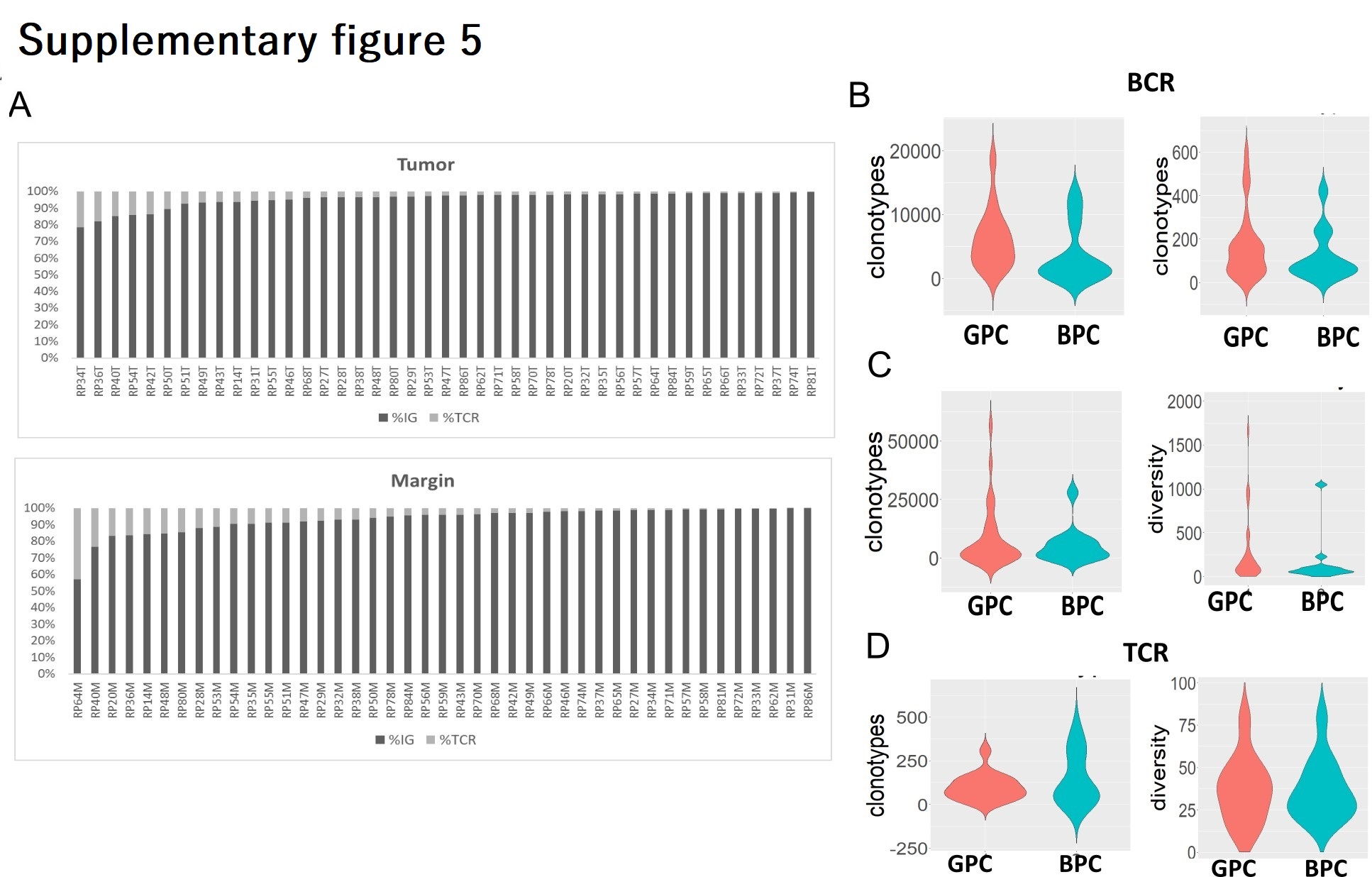

Supplement: Supplementary file 5 — Figure S5: [file CAM4-12-16774-s010.jpeg]

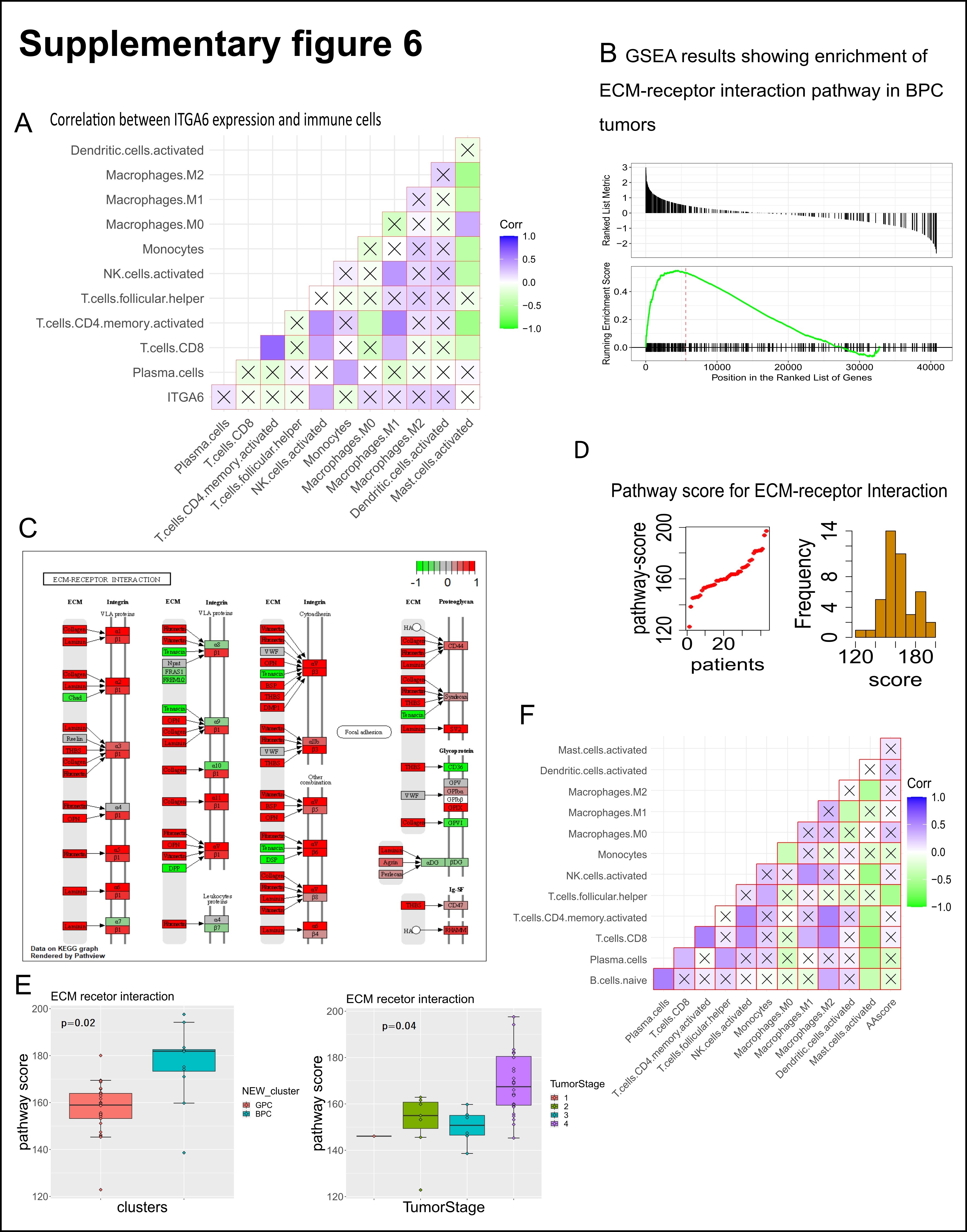

Supplement: Supplementary file 6 — Figure S6: [file CAM4-12-16774-s011.jpg]
