# Supplementary material for: Overexpression of CD73 is associated with recurrence and poor prognosis of gingivobuccal oral cancer as revealed by transcriptome and deep immune profiling of paired tumor and margin tissues
Source: Cancer Med. 2023 Jul 1;12(16):16774–87. doi: 10.1002/cam4.6299 (PMC10501293; doi:10.1002/cam4.6299)
Supplement: Supplementary file 11 — Data S1. [file CAM4-12-16774-s005.docx]

**Supplementary methods**

**Supplementary methods 1: RNA isolation and sequencing**

Approximately 25mg of tumor tissues were taken, stored in RNAlater overnight at 4degrees and then shifted to -80degrees for long term storage. RNA was extracted from the tissue samples using the RNeasy kit (QIAGEN) according to manufacturer’s protocol, and the quality was assessed using RNA Nano 6000 Assay Kit on Bioanalyzer 2100 system (Agilent Technologies). All isolated RNA samples were of high quality (OD260/OD280 ratio ≥ 2 and RNA Integrity Number (RIN) was ≥ 7). RNA sequencing libraries were generated using TruSeq RNA Library Prep Kit v2 (Illumina), following the manufacturer’s protocol. Libraries were sequenced at a target of 100 million reads per sample on Novaseq sequencer (Illumina) using 2 x 100 cycles. Raw sequencing reads were converted to FASTQ files; data quality was assessed using FASTQC (<https://qubeshub.org/resources/fastqc>). RNA-sequence data were processed, after removal of adapter sequences and low-quality reads, in accord with the TCGA mRNA Analysis pipeline (<https://docs.gdc.cancer.gov/Data/Bioinformatics_Pipelines/Expression_mRNA_Pipeline/>). Mapping of sequencing reads to the human genome reference sequence hg19 (NCBI 37) and post-mapping assembly of reads were done using the STAR (version 2.5.1a; <http://code.google.com/p/rna-star/>) package. Reads that mapped to multiple genomic locations and non-concordant reads were removed. Gene counts were determined using the HTSeq tool. Normalization of reads and test of equality of the levels of expression of genes between groups, after adjusting for differences in depth, were done using the DESeq2 Bioconductor package within the R statistical programming environment (1).

## **Supplementary methods 2: Identification of immune contexture and sub-grouping of patients**

Deconvolution of the expression matrix to estimate the proportions of immune cell types in the samples, after gene length normalization, was done using CIBERSORT (2) (<https://ciberfort.stanford.edu/>). The LM22 gene signature was used to estimate, using 1000 permutations, the proportions of 22 human immune cell types among the samples. Immune activity in the tumor was assessed by curating a list of 2039 immune-related genes (IRGs) from five different sources (CRIatlas, MSigDB C7 signature (www.gsea-msigdb.org/gsea/msigdb/), InnateDB (3), Immport (www.immport.org), and literature survey (4,5)). These genes are predominantly expressed by the immune cells or by cells known to be related to immune function.

# **Supplementary methods 3: Immunohistochemistry**

From each tumor tissue block, 3 μm-thick sections were prepared and dried in a 60˚C oven for 30 minutes. Single-marker immunohistochemistry (IHC) was performed in a Bond Max Automated Immuno-histochemistry Vision Bio-system (Leica Microsystems GmbH, Wetzlar, Germany) using standard protocols (27). Briefly, the sequential steps in IHC can be summarized as follows: antigen retrieval, addition of primary antibody, application of a secondary antibody that binds the primary antibody, and addition of a detection reagent to localize the primary antibody The immune markers used were CD20, CD3, CD4, CD8, CD45RA, CD45RO, GranzymeB and CD73. Digital images of the stained slides were captured using the Aperio Versa 8 platform (Leica, Wetzlar Germany). Images were captured at 20x magnification and analyzed using QuPath software. The immune cells were measured at 2 regions, namely the invasive margins and tumor centre (Reference given below #) and the average of the 2 regions was considered as tumor infiltrating lymphocytes. The level of an immune cell type was determined by the percentage of cells with a minimal intensity that was considered positive by two pathologists (A.C. and G.M). In a small number of cases where scoring seemed to be inaccurate due to mild background staining (5%), the slides were checked manually by two pathologists and the average score of their independent assessment was accepted (Supplementary file 2). Outlier detection was done using Dixon’s method, as implemented in the online package https://contchart.com/outliers.aspx. Further statistical analyses were done after outlier removal.

**Supplementary File 2**

**ANTIBODY DETAILS**

| Antibody | Clone | Dilution | Company |
| --- | --- | --- | --- |
| CD3 | F7.2.38 | RTU (Ready to Use) | DAKO |
| CD4 | EP 204 | RTU | PathnSitu |
| CD8 A | CD8/468 | RTU | PathnSitu |
| CD45RA | SPM504 | RTU | Master Diagnostics |
| CD45RO | UCHL-1 | RTU | PathnSitu |
| Granzyme B | M7235 | 1:50 | DAKO |
| CD20 | L26 | RTU | PathnSitu |
| CD73 | EPR6114 | 1:100 | Abcam |
|  |  |  |  |

**QUPATH QUANTIFICATION STRATEGY**

The IHC-based scoring methods were designed in QuPath software to segment cells based on staining intensity. The immune markers at the tumor invasive margins (IM) and tumor centre (CT) were analyzed.

**Digitization of the IHC slides:** The whole slide images were scanned using an automated bright field microscope (Aperio Versa 8, Leica, Germany) under 20x plan objective (NA 0.55, with final magnification × 100) with a resolution of 0.275 μm per pixel. The images were digitized to a pixel range of 1388 × 1040 pixels using CMOS camera. The image grabbing and preprocessing software package was inbuilt into the support of Aperio ImageScope (v12.3.3.5048) software platform.

**Image processing & analysis:** The selected whole slide images were annotated with disease grading, staging and other clinicopathological parameters by the expert oncopathologists. The resolution of the images was 2048 X 2048 with pixel size of 5.5 µm. The images were extracted and converted into tif file from scn extension file for further image analysis. Image processing and analysis was performed using Qupath (0.1.2). Selected tiff. files were opened in QuPath and following work flow followed:

1. Set image type: Brightfield (H-DAB) was selected

2. Set color deconvolution stain: Stain 1= Hematoxylin; Stain 2= DAB

3. Positive cell detection: In this step various parameters were defined for cell segmentation and quantification.

Table 1

| Parameter | Value |
| --- | --- |
| Detection image brightfield | Optical density (OD) sum |
| Requested pixel size microns | 0.5 |
| Background radius microns | 8.0 |
| Median radius microns | 0.0 |
| Sigma microns | 1.5 |
| Min area microns | 10.0 |
| Max area microns | 400 |
| Threshold | 0.1 |
| Max background | 2.0 |
| Watershed post process | true |
| Exclude DAB | false |
| Cell expansion microns | 5.0 |
| Include nuclei | true |
| Smooth boundaries | true |
| Make measurement | true |
| Threshold compartment | Cell: DAB OD mean |
| Threshold positive 1 | 1.5 |
| Threshold positive 2 | 0.1878/0.4197/1.5 |
| Threshold positive 3 | 0.2201/0.2197/0.4197 |
| Single threshold | False |
| True= selected, False= not selected | |

Immuno-scoring for both IM and CT were performed for each selected biomarker using QuPath software. The percentage values of DAB positive cells out of hematoxylin positive cells were recorded. Tumour center (CT) area of ~500 µm distance from invasive margin (IM) was selected for scoring immune cells using brush tool and wand tool wherever applicable. To generate the percentage positive scores of the individual markers, thresholds were developed in Qupath software (as per Table 1).

# Hendry S, Salgado R, Gevaert T et al (2017) Assessing tumorinfltrating lymphocytes in solid tumors: a practical review for pathologists and proposal for a standardized method from the international immuno-oncology biomarkers working group: part 2: TILs in melanoma, gastrointestinal tract carcinomas, non-small cell lung carcinoma and mesothelioma, endometrial and ovarian carcinomas, squamous cell carcinoma of the head and neck, genitourinary carcinomas, and primary brain tumors. Adv Anat Pathol 24:311–335. https://doi.org/10.1097/PAP.0000000000000161

References

1. Love MI, Huber W, Anders S. Moderated estimation of fold change and dispersion for RNA-seq data with DESeq2. Genome Biol [Internet]. 2014;15:550. Available from: https://doi.org/10.1186/s13059-014-0550-8

2. Newman AM, Liu CL, Green MR, Gentles AJ, Feng W, Xu Y, et al. Robust enumeration of cell subsets from tissue expression profiles. Nat Methods [Internet]. 2015;12:453–7. Available from: https://doi.org/10.1038/nmeth.3337

3. Breuer K, Foroushani AK, Laird MR, Chen C, Sribnaia A, Lo R, et al. InnateDB: systems biology of innate immunity and beyond--recent updates and continuing curation. Nucleic Acids Res [Internet]. Nucleic Acids Res; 2013 [cited 2022 Sep 9];41. Available from: https://pubmed.ncbi.nlm.nih.gov/23180781/

4. Chen Y, Li ZY, Zhou GQ, Sun Y. An Immune-Related Gene Prognostic Index for Head and Neck Squamous Cell Carcinoma. Clin Cancer Res [Internet]. Clin Cancer Res; 2021 [cited 2022 May 12];27:330–41. Available from: https://pubmed.ncbi.nlm.nih.gov/33097495/

5. Zhao XT, Zhu Y, Zhou JF, Gao YJ, Liu FZ. Development of a novel 7 immune-related genes prognostic model for oral cancer: A study based on TCGA database. Oral Oncol [Internet]. Oral Oncol; 2021 [cited 2022 May 12];112. Available from: https://pubmed.ncbi.nlm.nih.gov/33220636/

6. Mukherjee G, Bag S, Chakraborty P, Dey D, Roy S, Jain P, et al. Density of CD3+ and CD8+ cells in gingivo-buccal oral squamous cell carcinoma is associated with lymph node metastases and survival. PLoS One [Internet]. PLoS One; 2020 [cited 2022 May 12];15. Available from: https://pubmed.ncbi.nlm.nih.gov/33211709/

7. Dixon WJ. Analysis of Extreme Values. Ann Math Stat. 1950;21:488–506.

**Supplementary figure legends:**

**Supplementary figure 1: Distribution of the proportions of four immune cells in the tumors with and without observed Worst Pattern of Invasion (WPOI): (A) CD8+ T cells, (B) Plasma cells, (C) CD4+ T cells and (D) M1 macrophages. “ns”= not significant.**

**Supplementary figure 2: Differentially expressed immune-related genes:** (A) Volcano plot showing differentially expressed genes in tumor compared to adjacent normal tissue samples (n=43). (B) PCA with the expression levels of 544 IRGs showed the separate distribution of tumor samples from the negative margin and adjacent normal tissue samples. (C) Pathway enrichment analysis with differentially expressed IRGs between tumor and negative margins.

**Supplementary figure 3: Data on immunohistochemistry of five immune markers in tumor samples (n=33):** Distribution of %positive cells in the GPC and BPC tumors, were represented using violin plots for (A) CD45+, (B) GranzymeB+, (C) CD4+, (D) CD8+, (E) CD20+. Representative images of IHC staining of tumor tissues showed for three markers under 20X magnification: (F) CD4+ cells, (G) CD8+ cells and (H) CD20+ cells.

**Supplementary figure 4:** Scatter plots showing correlations between the expression of *ITGA6* and *CD73* genes in the TCGA-HNSC cohort samples with CD8+ T cells (A,B) and plasma cells (C,D).

**Supplementary figure 5**: **Immune repertoire richness and diversity across the clusters**. (A) Estimated composition of immune repertoire, including the T cell receptor (TCR) and B cell receptor (BCR). (B) Violin plots showing the differences in the total number of unique clonotypes in the tumors of GPC and BPC. (C) Violin plots showing the differences in the total number of unique clonotypes and repertoire diversity in the negative margins of GPC and BPC.

**Supplementary figure 6: (A**) Correlation between ITGA6 gene expression and proportions of immune cells in the tumors, (B) GSEA showing enrichment of “ECM-receptor interaction pathway ” among the BPC tumors compared to GPC tumors. (C) Overexpression of genes in the ECM-receptor interaction pathway in the BPC tumors compared to GPC tumors (pathway map taken from KEGG data base) (D) Estimated pathway specific score for ECM receptor interaction pathway. (E) Distribution of pathway specific scores for ECM receptor interactions among : (B) GPC and BPC tumors, (C) tumor stages. (F) Correlations between the immune cell types and the pathway-specific score.
